# *Lithocarpus polystachyus* Rehd. leaves aqueous extract inhibits learning and memory impairment in Alzheimer's disease rats: Involvement of the SIRT6/NLRP3 signaling pathway

**DOI:** 10.1002/ibra.12164

**Published:** 2024-06-03

**Authors:** Wendan Wu, You Yan, Tingting Yi, Yu Wei, Jianmei Gao, Qihai Gong

**Affiliations:** ^1^ Key Laboratory of Basic Pharmacology, Ministry of Education and Joint International Research Laboratory of Ethnomedicine Zunyi Medical University Zunyi China; ^2^ Key Laboratory of Basic Pharmacology of Guizhou Province, Department of Pharmacology, School of Pharmacy Zunyi Medical University Zunyi China; ^3^ Chinese Pharmacological Society‐Guizhou Province Joint Laboratory for Pharmacology Zunyi Medical University Zunyi Guizhou China; ^4^ Department of Neurology, The Affiliated Hospital of Zunyi Medical University Zunyi China

**Keywords:** Alzheimer's disease, *Lithocarpus polystachyus* Rehd., neuroinflammation, NLRP3 inflammasome, oxidative stress, SIRT6

## Abstract

Alzheimer's disease (AD) is a chronic and progressive neurodegenerative condition that is influenced by multiple factors along with neuroinflammation and oxidative stress. Our previous study proved that *Lithocarpus polystachyus* Rehd. aqueous extract (sweet tea aqueous extract, STAE) effectively inhibits hydrogen peroxide‐induced neuronal cell injury. However, it is not clear whether STAE can protect against AD, and its underlying mechanisms are still uncertain. Therefore, the present study was designed to evaluate the possible behavioral and neurochemical effects of STAE on A*β*_25‐35_‐induced AD rats administered STAE (20, 40, 80 mg/mL) for 14 days. We showed that STAE administration significantly and dose‐dependently ameliorated the cognitive deficits in the AD rat models, assessed in the Morris water maze (MWM) test, Y‐maze test, and novel object recognition (NOR) test. The results of hematoxylin and eosin (H&E) staining and Nissl staining showed that after treatment with STAE, the pathological damage to the hippocampal CA1, CA3, and dentate gyrus (DG) neurons of rats was significantly improved. Furthermore, STAE dose‐dependently inhibited microglia and astrocyte activation in the hippocampus of rats accompanied by increased protein expression of silent mating‐type information regulation 2 homolog 6 (SIRT6) and decreased protein expression of nod‐like receptor thermal protein domain‐associated protein 3 (NLRP3) and its downstream pyroptosis‐related genes after following A*β*_25‐35_. In summary, our findings indicate that STAE effectively inhibits A*β*_25‐35_‐induced learning and memory impairment in rats, and the mechanism is, at least partially, related to the regulation of SIRT6/NLRP3 signaling pathway.

## Graphical abstract

The findings of this study demonstrate that STAE effectively counteracts the learning and memory impairment induced by A*β*_25‐35_ in rats. This effect is attributed, at least in part, to the regulation of the SIRT6/NLRP3 signaling pathway, which inhibits neuroinflammation and oxidative stress. These results provide valuable insights into the potential therapeutic target of STAE for AD and offer a “proof‐of‐concept” for its efficacy in combating this disease.

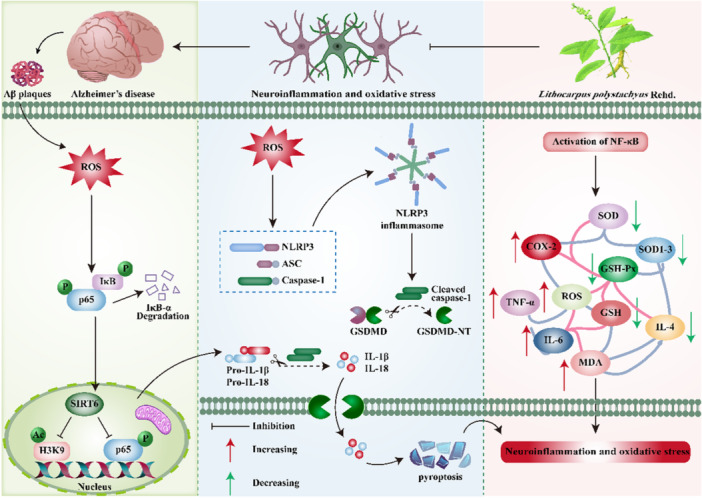

## 1 INTRODUCTION

Alzheimer's disease (AD) is a chronic and progressive neurodegenerative condition, accounting for an estimated 60%–70% of all dementia cases worldwide.^1^, ^2^ This condition is distinguished by the gradual decline in cognitive function, memory, and behavior, which is influenced by multiple factors.^3^ Its primary pathological alterations involve a deficiency in cholinergic activity, abnormal accumulation of amyloid protein, and excessive phosphorylation of Tau protein.^4^ Recent findings indicate that the deposition of amyloid‐*β* (A*β*) plays a crucial role in the early stages of AD pathogenesis, initiating a cascade of detrimental processes such as neuroinflammation, oxidative stress, and neuronal loss.^5^, ^6^ Neuroinflammation refers to the persistent inflammation occurring in the central nervous system (CNS), which is primarily caused by the activation of microglia and astrocytes resulting in the production of various pro‐inflammatory cytokines, including interleukin‐1*β* (IL‐1*β*), IL‐6, nitrate oxide (NO), and tumor necrosis factor‐*α* (TNF‐*α*).^7^ This inflammatory response leads to oxidative stress and significant neuronal damage.^8^ Oxidative stress, characterized by an imbalance in redox states within the body, involves the excessive generation of harmful reactive oxygen species (ROS) and/or dysfunction of the protective antioxidant system.^9^ ROS serves as a significant contributor to the initiation of neuroinflammation, whereby the subsequent inflammatory response can intensify ROS generation and exacerbate oxidative stress, thereby establishing a detrimental cycle.^10^ Thus, the interplay between neuroinflammation and oxidative stress assumes a pivotal role in the etiology and advancement of AD.^11^

Although the precise mechanism by which A*β* triggers neuroinflammatory and oxidative stress processes is intricate, there exists compelling evidence that the silent mating‐type information regulation 2 homolog 6 (SIRT6)/nod‐like receptor thermal protein domain‐associated protein 3 (NLRP3) signaling pathways are involved in the pathogenesis of AD.^12^, ^13^ SIRT6 exerts its anti‐inflammatory and antioxidative effects by reducing the histone H3 lysine 9 acetylation (H3K9Ac), thereby downregulating inflammatory factors and ROS levels through the nuclear factor‐*κ*B (NF‐*κ*B) pathway.^14^, ^15^ Activation of the NF‐κB pathway induces the expression of pro‐inflammatory cytokines and the formation of the NLRP3 inflammasome, a complex consisting of NLRP3, apoptosis‐associated speck‐like protein containing a caspase activation and recruitment domain (ASC), and cysteinyl aspartate‐specific proteinase‐1 (caspase‐1).^16^, ^17^, ^18^ It has been documented that microglia, astrocytes, and neurons express components of the NLRP3 inflammasome, which serves as a crucial target in neuroinflammation.^19^, ^20^ The activation of the NLRP3 inflammasome has been found to have multiple effects, including the promotion of inflammatory cytokine maturation and release, induction of cell pyroptosis, and triggering of severe inflammatory response and oxidative stress.^21^, ^22^ Consequently, the regulation of the SIRT6/NLRP3 pathway has proved to be a promising strategy to combat AD. It is worth noting that current anti‐AD drugs only provide relief for cognitive impairment symptoms without the ability to halt the progression of AD. Therefore, there is an urgent demand for the development of safer and more effective drugs.

*Lithocarpus polystachyus* Rehd. possess a distinctly sweet taste when infused with boiling water, leading to its designation as sweet tea (ST). This beverage has been consumed in China for over a millennium and has been utilized as both a medicinal tea and a traditional herbal remedy for the prevention and treatment of various ailments.^23^ Extensive research has revealed that ST aqueous extract (STAE) exhibits a wide range of pharmacological properties, including antioxidant,^24^ anti‐hyperglycemic,^25^ and anti‐hepatocyte death effects.^26^ Our previous study demonstrated that STAE exhibited neuroprotective effects on SH‐SY5Y cell injury induced by oxidative stress.^24^ Considering the available evidence on STAE's potential neuroprotective properties and its traditional use as a medicinal tea, we formulated the hypothesis that oral administration of STAE could confer a robust neuroprotective effect on neurological disorders. However, it remains unclear whether oral administration of STAE can provide protection against AD‐related neuroinflammation and oxidative stress induced by A*β*_25‐35_ and whether the SIRT6/NLRP3 signaling pathway is involved in the beneficial effects of STAE on AD. To address these uncertainties, AD rats induced by A*β*_25‐35_ were utilized to investigate the effect and the possible underlying mechanism of STAE on AD.

## 2 MATERIALS AND METHODS

### 2.1 Chemicals and reagents

The dried ST leaves were obtained from Fenggang City in Guizhou Province and identified by Professor Jianyong Zhang (School of Pharmacy, Zunyi Medical University, Zunyi, China). After being identified, it was confirmed that they belong to the dried leaves of *Lithocarpus polystachyus* Rehd., a plant in the family Fagaceae. Phlorizin and trilobatin were purchased from Chengdu Push Biotechnology Medical Technology Corporation. A*β*_25‐35_ (Cat #A4559) was purchased from Sigma‐Aldrich. Donepezil (DON; Cat #2107153) was purchased from Eisai China Inc. Toluidine blue (TB; Cat #G1436) was purchased from Solarbio. IL‐1*β* (Cat #RJ15465), interleukin‐4 (IL‐4; Cat #RJ15476), IL‐6 (Cat #RJ15478), IL‐10 (Cat #RJ15453), TNF‐*α* (Cat #RJ16622), transforming growth factor‐beta (TGF‐*β*; Cat #RJ16641), cyclooxygenase‐2 (COX‐2; Cat #RJ15770), NO (Cat #RJ16507), NO synthesis (NOS; Cat #RJ16698), interferon‐*γ* (IFN‐*γ*; Cat #RJ15676), ROS (Cat #RJ15780), superoxide dismutase (SOD; Cat #RJ16691), SOD1 (Cat #RJ28487), SOD2 (Cat #RJ28488), SOD3 (Cat #RJ29452), malondialdehyde (MDA; Cat #RJ15503), glutathione (GSH, RJ29397), and GSH peroxidase (GSH‐Px; Cat #RJ25745) assay kits were purchased from Shanghai Renjie Bioengineering Institute (Shanghai, China). Antibodies utilized against SIRT6 (Cat #ab191385), H3K9Ac (Cat #ab272150), Histone H3 (Cat #ab1791), NLRP3 (Cat #ab263899), IL‐18 (Cat #ab191860), caspase‐1 (Cat #ab286125), NF‐κB p65 (Cat #ab16502), phosphorylation‐NF‐κB p65 (p‐NF‐κB p65; Cat #ab76302), NF‐κB inhibitory protein (IκB‐*α*; Cat #ab32518), glial fibrillary acidic protein (GFAP; Cat #ab7260), and ionized calcium‐binding adapter molecule‐1 (Iba‐1; Cat #ab178847) were purchased from Abcam. Antibody utilized against cleaved‐caspase‐1 (Cat #AF4022) was purchased from Affinity Biosciences. Antibody utilized against ASC (Cat #abs155599) was purchased from Absin Bioscience Inc. Antibodies utilized against gasdermin D (GSDMD; Cat #20770‐1‐AP), *β*‐actin (Cat #66009‐1‐1 g), and *β*‐tubulin (Cat #662401‐lg) were purchased from Proteintech Group Inc.

### 2.2 STAE preparation and analysis

The dried leaves of ST were brewed in boiling water to make an ST infusion. The measured volume of double distilled water (ddH_2_O) was added to the weighed ST leaves, resulting in the formation of STAE. The containers were given a 30 min period of time to rest at ambient temperature. The aqueous extracts underwent filtration and cooling and were subsequently distributed into bottles. The raw material was mixed with 100 mL of ddH_2_O at 80°C, resulting in aqueous extracts of 20, 40, and 80 mg/mL, respectively. Fresh aqueous extracts were made every day.

Liquid chromatography‐mass spectrometry (LC‐MS) analysis was performed for quality control of the STAE utilizing Phenomenex Gemini C18 combined with a 4000 QTRAP mass spectrometer (SCIEX) at a column temperature of 30°C. The mobile phase, consisting of an aqueous solution containing 0.2% formic acid (A) and 0.2% formic acid in acetonitrile (B), was administered at a rate of 0.2 mL/min, with intervals of 0–3 min (80%–60% A), 3–4 min (60%–10% A), 4–5 min (10%–70% A), and 5–6 min (70% A).

### 2.3 Animal

Male Sprague Dawley rats (7–8 weeks, 260–280 g) were procured from Hunan SJA Laboratory Animal Co., Ltd. (Certificate number: SCXK (Xiang) 2019‐0004, Changsha, China). The animals were reared in cages, with a range of 5 to 6 and were subjected to a 12 h light–dark cycle, maintaining an optimal temperature (23 ± 1°C) and humidity (55% ± 5%), while being granted unrestricted access to water. The Experimental Animal Ethics Committee of Zunyi Medical University granted approval for all animal experimental operations (No. ZMU21‐2303‐333), and all animal care and experimental procedures were carried out in compliance with the National Institutes of Health guide for the care and utilization of laboratory animals (NIH Publications No. 8023, revised 1978).

### 2.4 Experimental designs

The rats were randomly allocated into seven groups (*n* = 15 per group): sham, sham + 80 mg/mL STAE (sham + STAE8), A*β*_25‐35_, A*β*_25‐35_ + 20 mg/mL STAE (A*β*_25‐35_ + STAE2), A*β*_25‐35_ + 40 mg/mL STAE (A*β*_25‐35_ + STAE4), A*β*_25‐35_ + 80 mg/mL STAE (A*β*_25‐35_ + STAE8), and A*β*_25‐35_ + DON (1 mg/kg). Owing to its superior clinical effectiveness, DON has emerged as the primary positive control medication in AD disease research, utilized to exclude false negative results. The AD animal model was built using intracerebroventricular (ICV) injection of A*β*_25‐35_, which is known to be a toxic part of full‐length A*β* and can be used to induce oxidative stress, microglial activation, and neuroinflammation.^27^ To summarize, the A*β*_25‐35_ was gathered by incubating it in normal saline (NS) at 37°C for 4 days and then diluted to its ultimate concentration with saline before the experiment. In short, the rats underwent an 8 h fast and were administered intraperitoneal sodium pentobarbital anesthesia (50 mg/kg), and small burr holes were drilled on both sides of the skull (1.0 mm posterior to bregma and 1.5 mm lateral to the midline) to allow ICV injection of A*β*_25‐35_ at the depth of 3.5 mm. The injection had a duration of 5 min, with the needle remaining in position for an additional 5 min following the injection. The sham group and sham + STAE8 group were subjected to the identical procedure as previously stated, without the introduction of A*β*_25‐35_. The experimental rats were orally administered freshly prepared STAE at varying doses of 20, 40, and 80 mg/mL starting from the second day after the neurosurgery for a duration of 14 days, followed by 11 days of behavioral tests. The ddH_2_O treatment was administered to both the sham group and the A*β*_25‐35_ group in accordance with their respective volumes. The A*β*_25‐35_ + DON group received a dosage of 1 mg/kg DON. The rats with administration of STAE were allowed free access to 200 mL STAE that was prepared fresh and handled daily, while other rats were access to the same volume of ddH_2_O. Every set of animals had the freedom to consume food and water without any restrictions.

### 2.5 Morris water maze (MWM) test

MWM test was conducted to assess the spatial learning and memory ability of the rats, as described in our prior study.^28^ In a nutshell, the experiment was conducted with a water maze that had a circular pool of 160 cm in diameter, 50 cm in height, and a water temperature of 23 ± 1°C. The pool area was split into four equal sections, with a platform (10 cm in diameter) submerged 1 cm beneath the water surface. Following a 6‐day navigation experiment to locate the platform that was submerged 1 cm beneath the water surface, a probe trial was carried out on the 7th day to assess memory retention. The analysis‐management system measured the escape latency, time spent in the target quadrant, number of target crossings, and swimming speed.

### 2.6 Y‐maze test

The Y‐maze test was employed to assess the spatial recognition memory of the rats, as outlined in our prior study.^29^ Succinctly put, the Y‐maze was executed utilizing a device featuring matching arms (measuring 50 cm × 17 cm × 30 cm). The rats were placed in a single starting arm and allowed to freely explore all three arms for a duration of 10 min, after which the numerical entries into each arm were documented. Spontaneous alternation (%) was employed as a metric to ascertain spatial recognition memory, with its value calculated as [(number of actual alternations)/(number of total arm entries − 2)] × 100. A high percentage of spontaneous alternation indicates a good spatial recognition memory and cognitive function.

### 2.7 New object recognition (NOR) test

NOR test was used to investigate the rats' ability to remember and recognize objects, as described in our earlier study.^30^ The apparatus was composed of a reaction box (50 cm × 50 cm × 50 cm), an automatic recording and analyzing system, a green square of object A (5 cm × 5 cm × 5 cm), and a red cone of object B (5 cm in diameter and 5 cm in height). In the first 2 days, two identical objects A are placed 10 cm away from the adjacent wall of the reaction chamber, and the rats were allowed to explore the two objects for 10 min. On the third day, one of the items was swapped out for object B, and the rats could freely explore for 10 min. The rats exhibited exploratory behavior by directing their nose and/or forepaws toward objects within a 2 cm radius or through physical contact. The discrimination index was used to determine the proportion of time dedicated to exploring the novel object in relation to the overall duration spent in both objects, with a higher discrimination index suggesting a better cognitive function.

### 2.8 Tissue collection

All rats were given intraperitoneal sodium pentobarbital anesthesia (50 mg/kg) after the behavioral tests, and samples were collected. First, we collected blood from the abdominal aorta, which was put into a centrifuge for 3000 × g (4°C, 15 min) to separate the serum and then stored in a refrigerator at −20°C for enzyme‐linked immunosorbent assay (ELISA). Then, three rats in each group were given cardiac perfusion with phosphate buffer (PBS, 0.1 mol/L, pH 7.4) followed by 4% paraformaldehyde. Subsequently, the brains were removed immediately after perfusion and placed in 4% paraformaldehyde fixative for more than 72 h and subjected to paraffin embedding technique. The brains were sectioned (4–5 μm thickness) using a Leica vibrating blade microtome for histopathological analysis. At last, the remaining rats were decapitated immediately, and their hippocampal tissue was quickly isolated and frozen at −80°C for western blot (WB) analysis.

### 2.9 Hematoxylin and eosin (H&E) staining

H&E staining was used to estimate the morphological alterations of hippocampal neurons in rats. In brief, after routine dewaxed and rehydrated, the sections of brain tissue were stained with hematoxylin solution for 15 min and then washed under running water for 1 min. After that, the sections were immersed in eosin solution for 4 min. After sections were dried, they were sealed with neutral gum. A light microscope (BX 43 Olympus) was used to observe the morphological changes of hippocampal regions (CA1, CA3, DG).

### 2.10 Nissl staining

Nissl staining was used to assess the survival neurons of the hippocampus in rats. Succinctly put, after routine dewaxing and rehydration, the sections of brain tissue were immersed in the TB stain solution at 37°C for 30 min. The slides were rinsed in ddH_2_O and then sealed with neutral gum. The prepared sections were examined using a light microscope (BX 43 Olympus). The survival neurons of hippocampal regions (CA1, CA3, DG) were analyzed using ImageJ software. Neurons possessing unaltered Nissl bodies are classified as surviving neurons by ImageJ software and as indicators of pathological structural alterations.

### 2.11 Immunofluorescence (IF) staining

The proliferation of microglia and astrocyte was identified through Iba‐1 staining and GFAP staining using IF staining. Briefly, after routine dewaxing and rehydration, the sections of brain tissue were repaired by microwave heating in sodium citrate buffer (0.1 mol/L, pH 6.0) for 18 min and then blocked with 10% goat serum for 45 min. Next, the primary antibodies monoclonal rabbit anti‐Iba‐1 (1:100) and monoclonal rabbit anti‐GFAP (1:200) were applied to the slices overnight at 4°C, followed by a 40 min incubation with goat anti‐rabbit IgG (1:200) at 37°C. After washing with PBS, the sections were restained with 4′,6‐diamidino‐2‐phenylindole (DAPI) for 10 min. Each rat hippocampal CA1, CA3, and DG sections were examined under a light microscope (BX 53 Olympus) to determineimmunoreactivity. The target protein immunoreactivity was measured using ImageJ software and normalized. The threshold was established and standardized across images to maximize the true protein expression signal for quantification. The percentage of positive area was calculated: positive area % = (positive area of the target protein/positive area of the DAPI) × 100%.

### 2.12 ELISA

ELISA was used to determine the levels of inflammatory factors and redox status in the serum of rats, as described previously.^31^ The levels of IL‐1*β*, IL‐4, IL‐6, IL‐10, COX‐2, TGF‐*β*, NO, NOS, TNF‐*α*, IFN‐*γ*, ROS, and MDA and the activities of SOD, SOD1, SOD2, SOD3, GSH, and GSH‐Px were assessed according to the specified ELISA kits. The optical density at 450 nm of each well was measured. The concentration of the target protein in the samples was determined by comparing the absorbance values to the standard curve generated from known concentrations of the target protein.

### 2.13 WB analysis

WB analysis was carried out in accordance with the previously outlined procedure.^32^ In brief, RIPA buffer was used to homogenize the hippocampal tissues of rats. Subsequently, an equivalent quantity of protein was isolated using sodium dodecyl sulfate‐polyacrylamide gel electrophoresis and subsequently transferred onto a polyvinylidene fluoride (PVDF) membrane. Following that, the membrane was blocked with 5% nonfat milk in TBST at room temperature for a duration of 3 h. Subsequently, the membranes were subjected to incubation with corresponding primary antibodies, including NLRP3 (1:1000), ASC (1:1000), GSDMD (1:1000), caspase‐1 (1:1000), cleaved‐caspase‐1(1:1000), p‐NF‐κB p65 (1:1000), NF‐κB p65 (1:1000), I*κ*B‐*α* (1:2000), SIRT6 (1:2000), H3K9Ac (1:1000), histone H3 (1:1000), and IL‐18 (1:1000), overnight at 4°C. Subsequently, membrane‐bound antibodies were labeled using a species‐specific HRP‐conjugated secondary antibody, with *β*‐actin, *β*‐tubulin, or Histone H3 serving as a loading control. Representative bands were visualized using ECL Western blot detection reagents, and images were collected and then used for the final protein expression assays using Image J software and normalized to loading controls. Protein expression levels were calculated as relative expression = optical density value of target protein/to optical density value of *β*‐actin/*β*‐tubulin/histone H3.

### 2.14 Data and statistical analysis

All data were expressed as the mean ± standard deviation (SD) and analyzed using SPSS 29.0 (SPSS, Inc.). Two or multiple groups were compared using Student's unpaired *t* test or one‐way ANOVA, respectively. For the MWM test, two‐way repeated‐measures ANOVA was utilized. Bonferroni's post hoc tests were run only when *F* achieved *p* < 0.05 and there was no significant variance in homogeneity. *p* < 0.05 was set as indicating statistical significance.

## 3 RESULTS

### 3.1 Preparation and analysis of STAE

**Figure 1:**
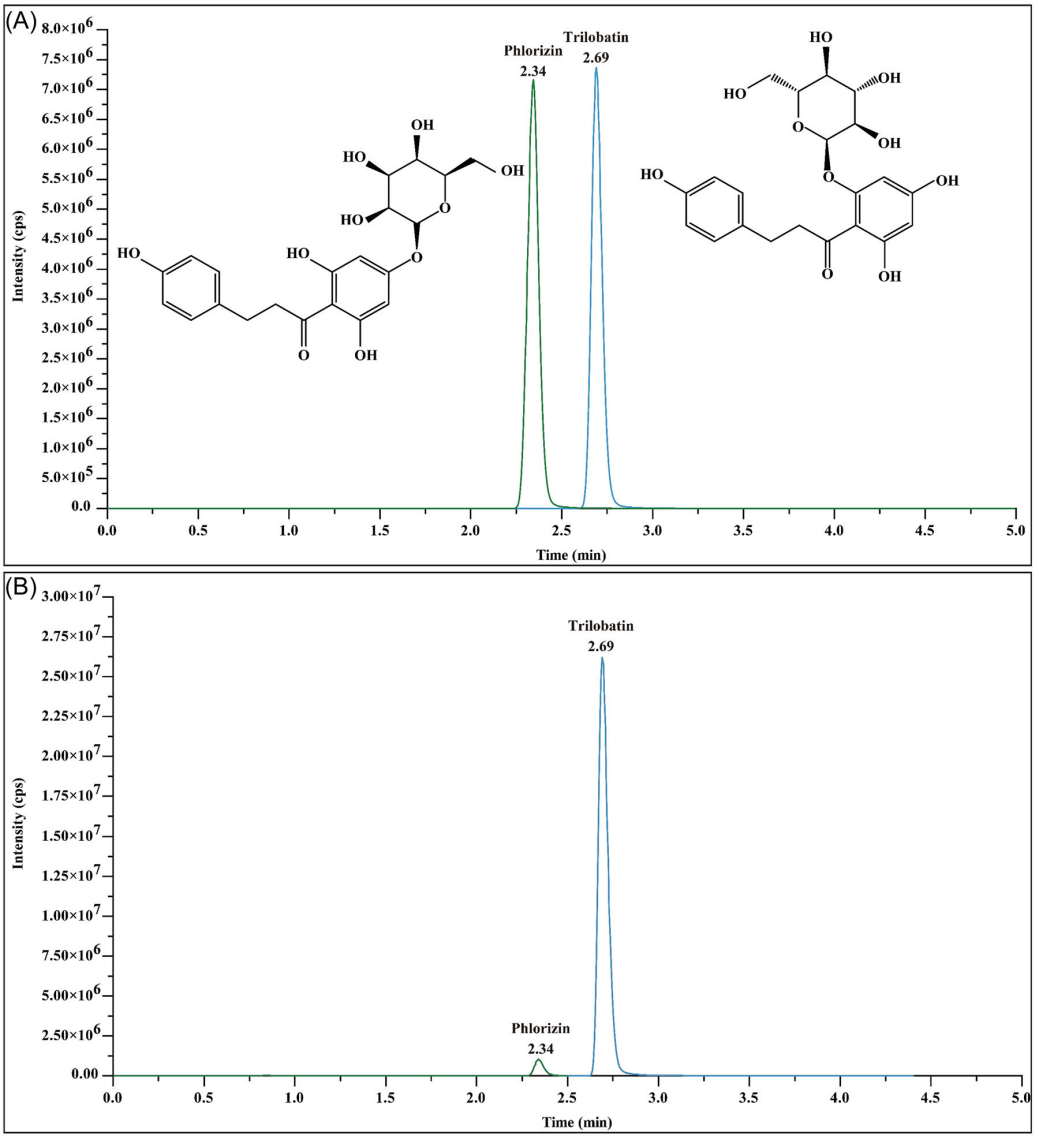
Analysis of main components of sweet tea aqueous extract (STAE). (A) Ion chromatograms of trilobatin and phlorizin standard samples. (B) Ion chromatogram of STAE. [Color figure can be viewed at wileyonlinelibrary.com]

The composition of STAE, which was derived from dried ST leaves, was examined using LC‐MS following the aforementioned procedure. The chromatograms of STAE obtained through LC‐MS analyses displayed two conspicuous peaks (Figure 1A,B). The major constituents of STAE were identified as both trilobatin and phlorizin among the peaks that were detected.

### 3.2 STAE rescued cognitive impairment in A*β*_25‐35_‐induced AD rats

**Figure 2:**
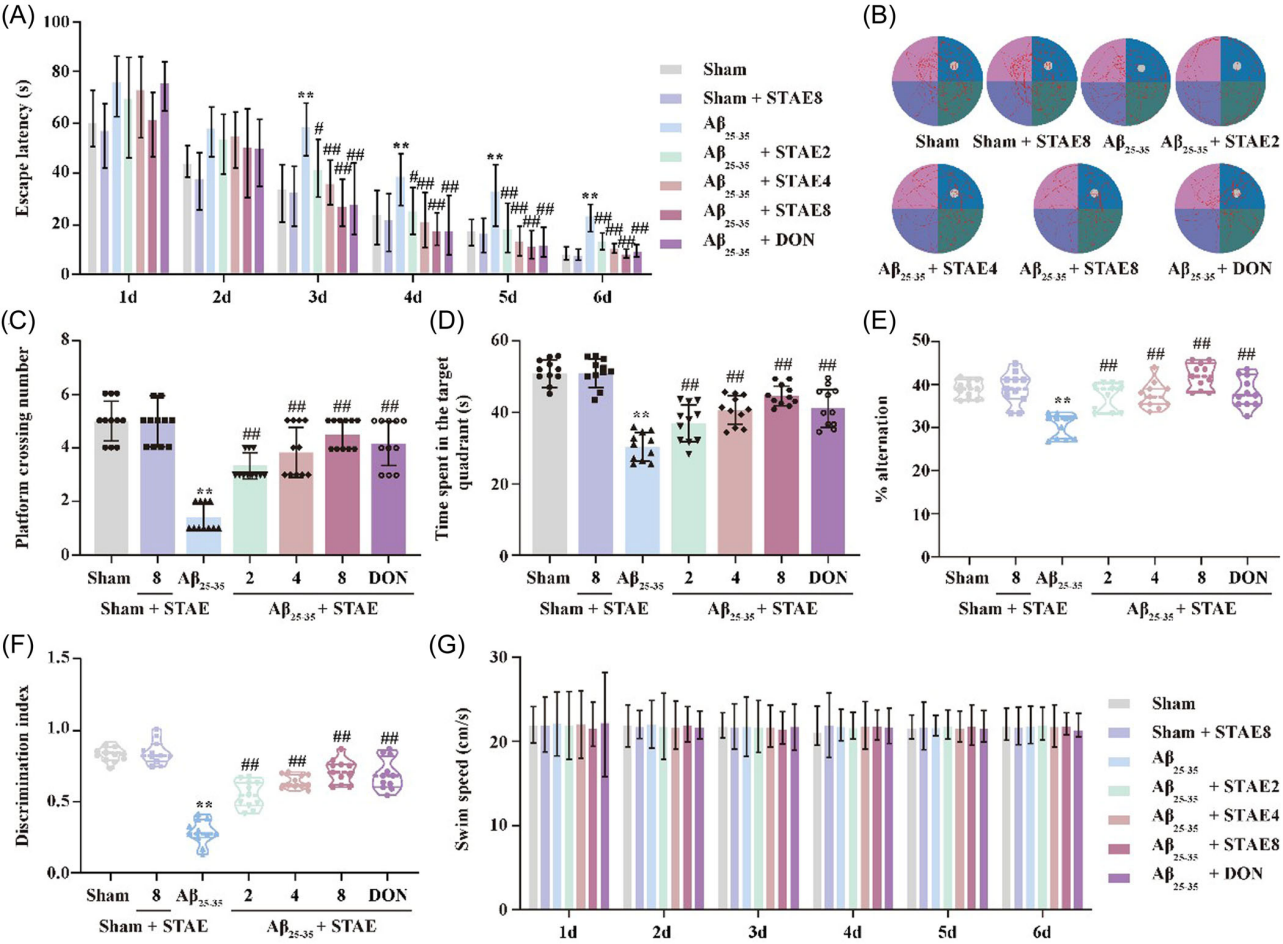
Sweet tea aqueous extract (STAE) rescued cognitive impairment in A*β*_25‐35_‐induced Alzheimer's disease (AD) rats. (A) Escape latency (*n* = 11). (B) Representative track images of rats in the probe test. (C) The number of crossing platforms in the probe test (*n* = 11). (D) Time spent in the quadrant (*n* = 11). (E) Alternation rates (%) in the Y‐maze task (*n* = 11). (F) Discrimination index in the novel object recognition (NOR) test (*n* = 11). (G) Swimming speed (*n* = 11). STAE, *Lithocarpus polystachyus* Rehd. aqueous extract; sham, sham group; sham + STAE8, sham group administrated with 80 mg/mL STAE; A*β*_25‐35_, A*β*_25‐35_ group; A*β*_25‐35_ + STAE2, A*β*_25‐35_ group administrated with 20 mg/mL STAE; A*β*_25‐35_ + STAE4, A*β*_25‐35_ group administrated with 40 mg/mL STAE; A*β*_25‐35_ + STAE4, A*β*_25‐35_ group administrated with 80 mg/mL STAE; A*β*_25‐35_ + DON, A*β*_25‐35_ group administrated with 1 mg/kg Donepezil. The data were expressed as the mean ± SD. *p < 0.01 versus sham group; ^*#*^ *p* < 0.05, ^*##*^ *p* < 0.01 versus A*β*_25‐35_ group. [Color figure can be viewed at wileyonlinelibrary.com]

To comprehend the impact of STAE on cognitive decline, AD rats induced by A*β*_25‐35_ were employed. First, MWM test was used to assess the spatial learning and memory of rats. Compared with the sham group, A*β*_25‐35_ rats exhibited notable deficits in spatial learning and memory, as indicated by extended escape latency time during the training trails, a reduced number of crossings on the platform, and a shorter duration spent in the target quadrant of the probe trail. Nevertheless, the administration of STAE treatment effectively rectified these abnormalities in AD rats (Figure 2A–D). Additionally, A*β*_25‐35_‐treated rats exhibited a decrease in both the percentage of correct spontaneous alternations during the Y‐maze test and the discrimination index between novel and familiar objects in NOR test, whereas STAE effectively counteracted these alterations (Figure 2E,F), suggesting that STAE successfully mitigated cognitive impairment in AD rats. Furthermore, there was no change in the swimming speed among all groups throughout the training period, indicating that the rats treated with A*β*_25‐35_ did not experience any motor impairment (Figure 2G). Collectively, these findings demonstrate the efficacy of STAE in alleviating cognitive impairments in AD rats.

### 3.3 STAE effectively ameliorated neuronal pathological alteration hippocampus induced by A*β*_25‐35_ in rats

**Figure 3:**
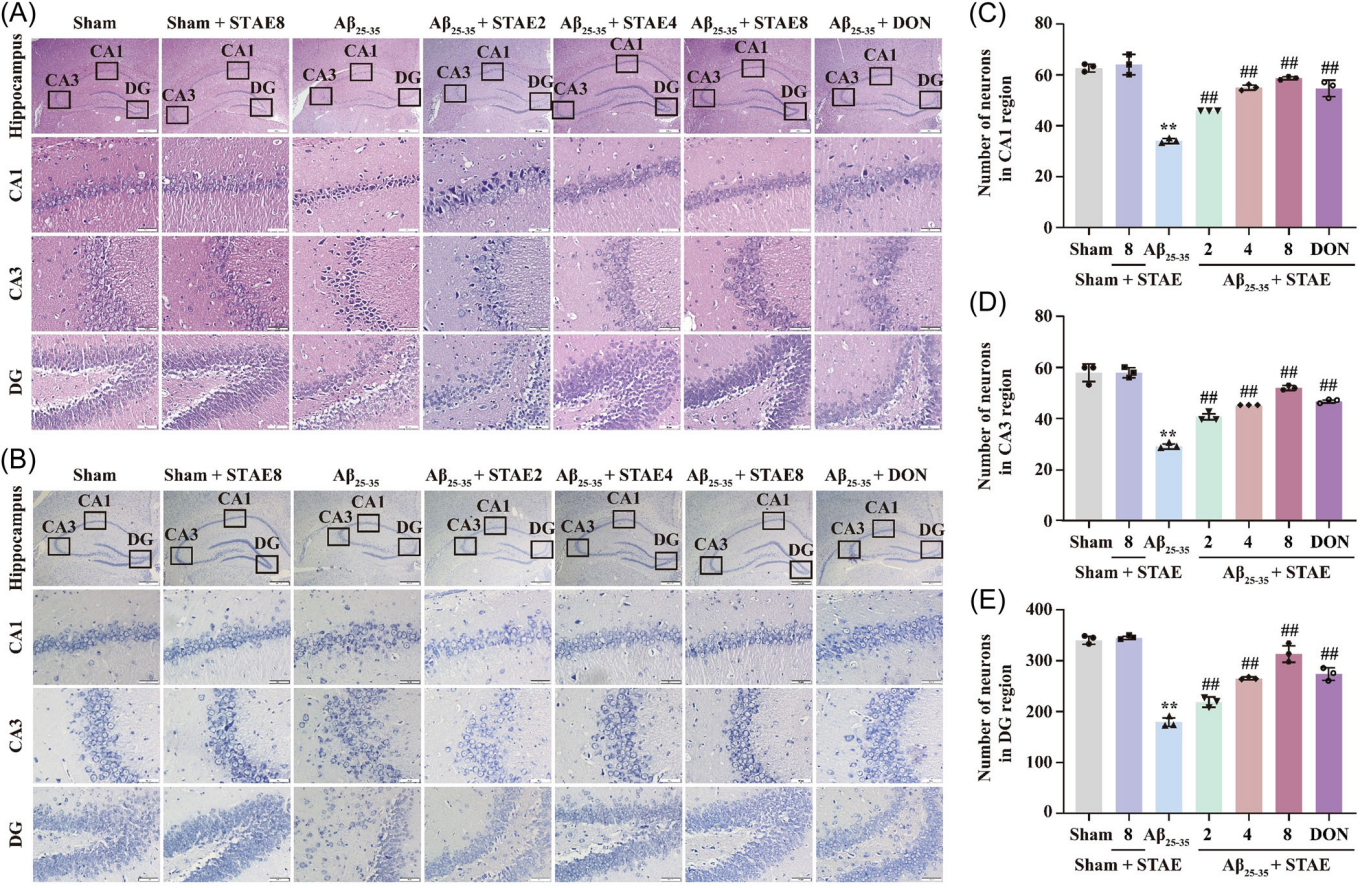
Sweet tea aqueous extract (STAE) improved pathological injury in the hippocampus of A*β*_25‐35_‐induced Alzheimer's disease (AD) rats. (A) The typical representative pictures of H&E staining in the hippocampus. (B) The typical representative pictures of Nissl staining in the hippocampus. (C) Statistical analysis of the number of surviving neurons in the CA1 region (*n* = 3). (D) Statistical analysis of the number of surviving neurons in the CA3 region (*n* = 3). (E) Statistical analysis of the number of surviving neurons in the dentate gyrus (DG) region (*n* = 3). STAE, *Lithocarpus polystachyus* Rehd. aqueous extract; sham, sham group; sham + STAE8, sham group administrated with 80 mg/mL STAE; A*β*_25‐35_, A*β*_25‐35_ group; A*β*_25‐35_ + STAE2, A*β*_25‐35_ group administrated with 20 mg/mL STAE; A*β*_25‐35_ + STAE4, A*β*_25‐35_ group administrated with 40 mg/mL STAE; A*β*_25‐35_ + STAE4, A*β*_25‐35_ group administrated with 80 mg/mL STAE; A*β*_25‐35_ + DON, A*β*_25‐35_ group administrated with 1 mg/kg donepezil. The data were expressed as the mean ± SD. **p < 0.01 versus sham group; ^##^ *p* < 0.01 versus A*β*_25‐35_ group. Magnification: 40× (upper panel), 400× (lower panel); bar scale: 500 µm (upper panel), 50 µm (lower panel). [Color figure can be viewed at wileyonlinelibrary.com]

To assess the effect of STAE on neuronal damage caused by A*β*_25‐35_, we used H&E staining and Nissl staining. The results of H&E and Nissl staining for pathological damage and neuronal survival showed that compared with the sham group, A*β*_25‐35_‐treated rats had significant pathological damage in the hippocampal regions (CA1, CA3, DG), including loose cell arrangement, incomplete structure, uneven staining, and reduced number of Nissl bodies and cells. However, after treatment with STAE, the pathological damage to the hippocampal regions (CA1, CA3, DG) (Figure 3A) and neuron loss in rats were significantly improved (Figure 3B–E). These results suggest that STAE can effectively improve the loss of hippocampal neurons in AD rats.

### 3.4 STAE repressed A*β*_25‐35_‐induced neuroinflammation and oxidative stress via regulating SIRT6/NLRP3 signaling pathway and downregulated NLRP3 downstream pyroptosis‐related genes

**Figure 4:**
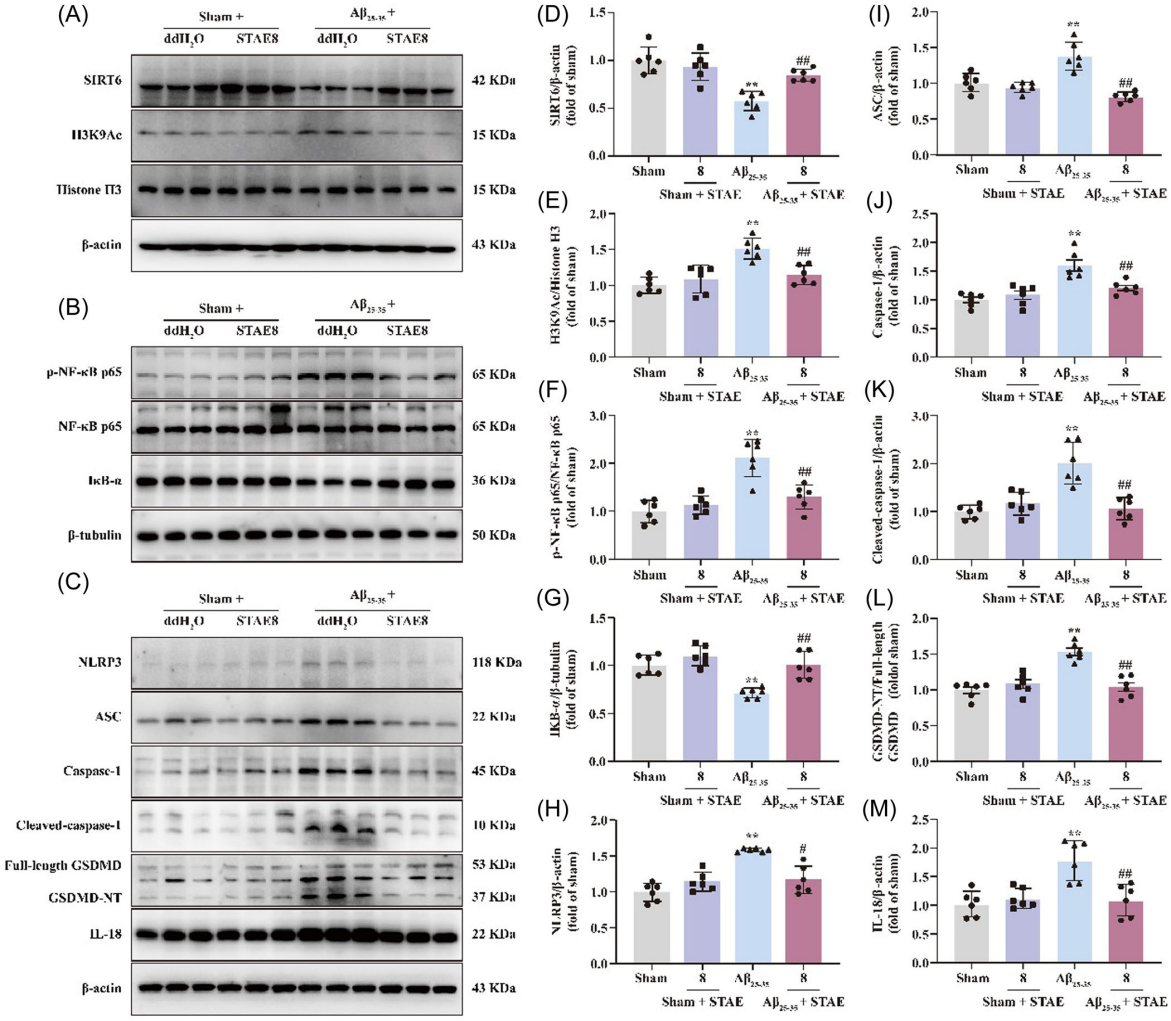
Sweet tea aqueous extract (STAE) activated the SIRT6/NLRP3 signaling pathway in A*β*_25‐35_‐induced Alzheimer's disease (AD) rats. (A) Representative Western blot of SIRT6, H3K9Ac protein expression. (B) Representative Western blot analysis of p‐NF‐κB p65 protein level and I*κ*B‐*α* protein expression. (C) Representative Western blot analysis of NLRP3, ASC, caspase‐1, cleaved‐caspase‐1, GSDMD‐NT, and IL‐18 protein expression. (D) Quantification of SIRT6 protein expression in the hippocampus (*n* = 6). (E) Quantification of H3K9Ac protein expression in the hippocampus (*n* = 6). (F) Quantitation of p‐NF‐κB p65 protein level in the hippocampus (*n* = 6). (G) Quantification of I*κ*B‐*α* protein expression in the hippocampus (*n* = 6). (H) Quantification of NLRP3 protein expression in the hippocampus (*n* = 6). (I) Quantification of ASC protein expression in the hippocampus (*n* = 6). (J) Quantification of caspase‐1 protein expression in the hippocampus (*n* = 6). (K) Quantification of cleaved‐caspase‐1 protein expression in the hippocampus (*n* = 6). (L) Quantification of GSDMD‐NT protein expression in the hippocampus (*n* = 6). (M) Quantification of IL‐18 protein expression in the hippocampus (*n* = 6). STAE, *Lithocarpus polystachyus* Rehd. aqueous extract; sham, sham group; sham + STAE8, sham group administrated with 80 mg/mL STAE; A*β*_25‐35_, A*β*_25‐35_ group; A*β*_25‐35_ + STAE2, A*β*_25‐35_ group administrated with 20 mg/mL STAE; A*β*_25‐35_ + STAE4, A*β*_25‐35_ group administrated with 40 mg/mL STAE; A*β*_25‐35_ + STAE4, A*β*_25‐35_ group administrated with 80 mg/mL STAE; A*β*_25‐35_ + DON, A*β*_25‐35_ group administrated with 1 mg/kg donepezil. The data were expressed as the mean ± SD (*n* = 6). **p < 0.01 versus sham group; ^#^ *p* < 0.05, ^##^ *p* < 0.01 versus A*β*_25‐35_ group. [Color figure can be viewed at wileyonlinelibrary.com]

In addition, WB was employed to ascertain the expression of the essential proteins in the SIRT6/NLRP3 signaling pathway and its associated pyroptosis‐related genes. The A*β*_25‐35_‐treated rats exhibited significantly higher levels of NLRP3, ASC, caspase‐1, cleaved‐caspase‐1, GSDMD‐NT, H3K9Ac, IL‐18 protein expressions, and p‐NF‐κB p65 compared to the sham group, while SIRT6, I*κ*B‐*α* protein expressions showed a decrease in the A*β*_25‐35_‐treated group compared to the sham group; conversely, STAE effectively reversed these alterations (Figure 4A–M). The results imply that STAE mitigates neuroinflammation and oxidative stress, to some extent, by regulating the SIRT6/NLRP3 signaling pathway.

### 3.5 STAE inhibited activation of microglia and astrocytes in the hippocampus of AD rats

**Figure 5:**
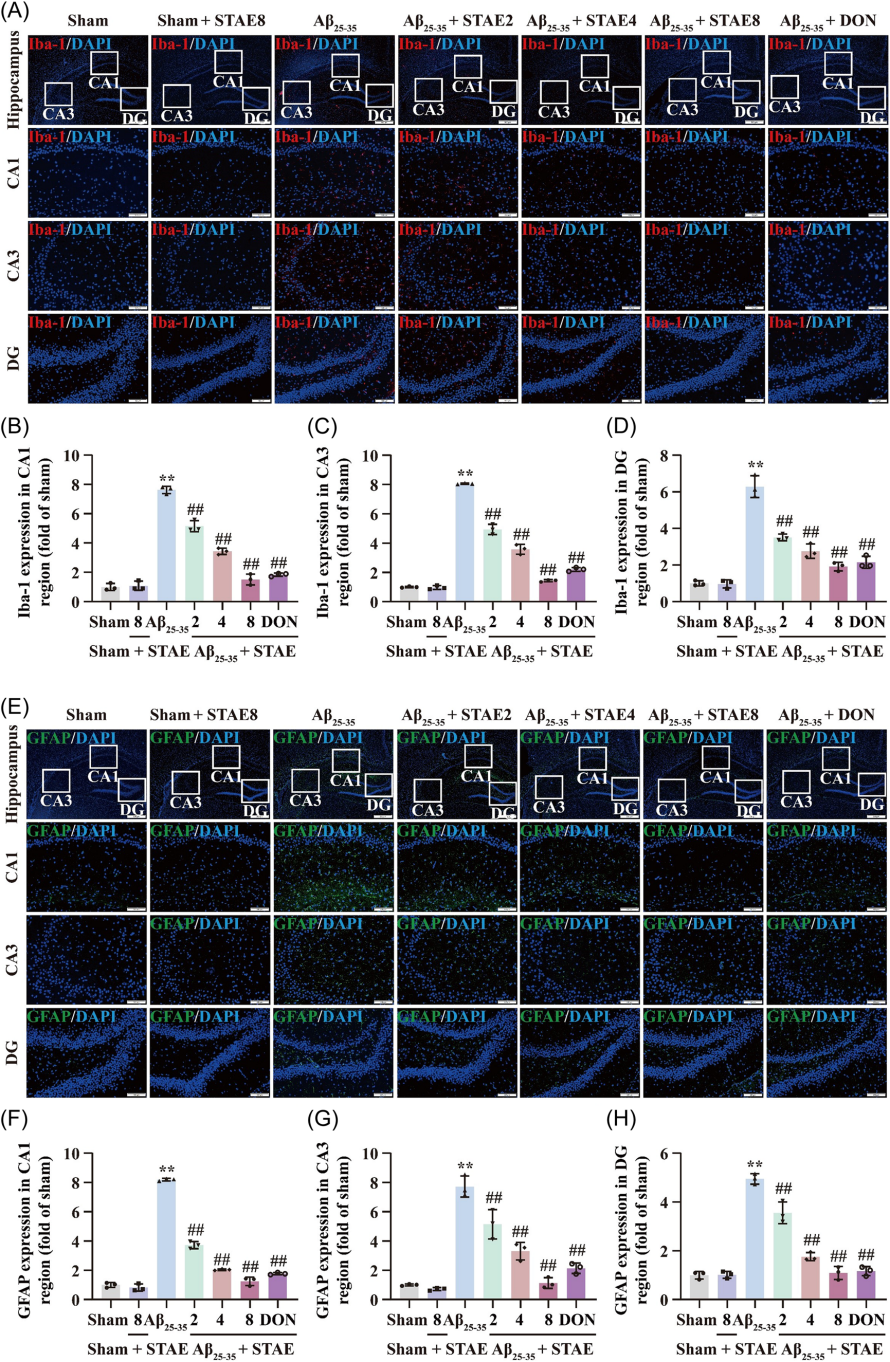
Sweet tea aqueous extract (STAE) inhibited activation of microglia and astrocyte in the hippocampus of A*β*_25‐35_‐induced Alzheimer's disease (AD) rats. (A) Representative images of Iba‐1 expression (red) and 4′,6‐diamidino‐2‐phenylindole (DAPI; blue) in the hippocampus. (B) Iba‐1 expression in the CA1 region (fold of sham) (*n* = 3). (C) Iba‐1 expression in the CA3 region (fold of sham) (*n* = 3). (D) Iba‐1 expression in the dentate gyrus (DG) region (fold of sham) (*n* = 3). (E) Representative images of glial fibrillary acidic protein (GFAP) expression in the hippocampus. (F) GFAP expression in the CA1 region (fold of sham) (*n* = 3). (G) GFAP expression in the CA3 region (fold of sham) (*n* = 3). (H) GFAP expression in the DG region (fold of sham) (*n* = 3). STAE, *Lithocarpus polystachyus* Rehd. aqueous extract; sham, sham group; sham + STAE8, sham group administrated with 80 mg/mL STAE; A*β*_25‐35_, A*β*_25‐35_ group; A*β*_25‐35_ + STAE2, A*β*_25‐35_ group administrated with 20 mg/mL STAE; A*β*_25‐35_ + STAE4, A*β*_25‐35_ group administrated with 40 mg/mL STAE; A*β*_25‐35_ + STAE4, A*β*_25‐35_ group administrated with 80 mg/mL STAE; A*β*_25‐35_ + DON, A*β*_25‐35_ group administrated with 1 mg/kg Donepezil. The data were expressed as the mean ± SD. **p < 0.01 *versus* sham group, ^##^ *p* < 0.01 versus A*β*_25‐35_ group. Magnification: 40 × (upper panel), 200 × (lower panel); bar scale: 500 µm (upper panel), 100 µm (lower panel). [Color figure can be viewed at wileyonlinelibrary.com]

Subsequently, to investigate the effects of STAE on the activation of microglia and astrocytes in the hippocampus of AD rats, we conducted IF staining to observe the presence of the microglia marker (Iba‐1) and the astrocyte marker (GFAP) in the CA1, CA3, and DG hippocampal regions. The A*β*_25‐35_‐treated rats exhibited a substantial rise in Iba‐1 expressions in the CA1, CA3, and DG hippocampal regions compared to the sham group. Nevertheless, a notable decline in Iba‐1 expressions was noted in the CA1, CA3, and DG hippocampal regions of the STAE group compared to the A*β*_25‐35_ group (Figure 5A–D). Interestingly, astrocytes exhibit a consistent activation pattern with microglia (Figure 5E–H), indicating that STAE effectively suppressed the activation of microglia and astrocyte in the hippocampus of AD rats induced by A*β*_25‐35_. The results suggest that STAE has the capability to cease neuroinflammation during the initial phase of AD.

### 3.6 STAE represses A*β*_25‐35_‐induced neuroinflammation and oxidative stress

**Figure 6:**
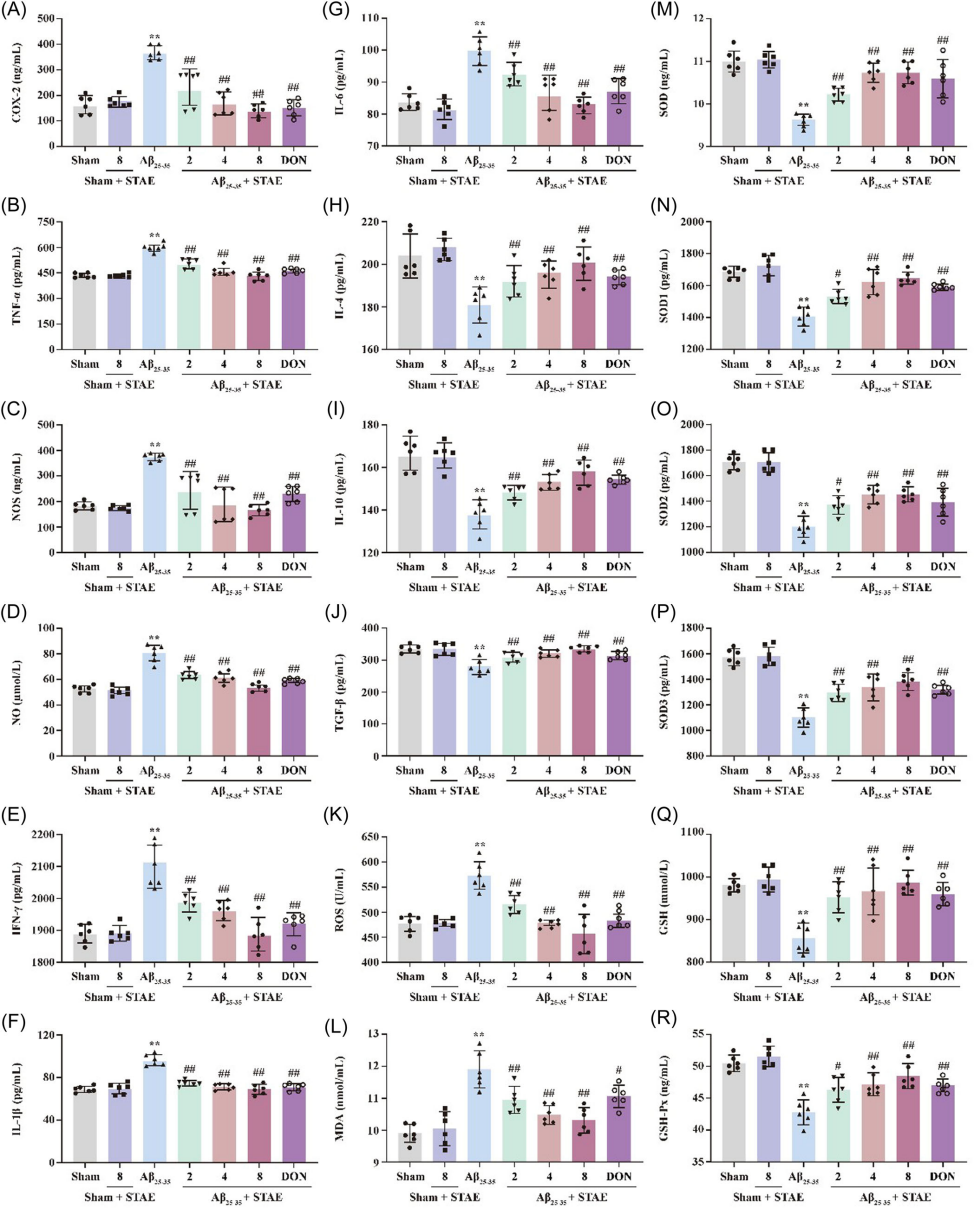
Sweet tea aqueous extract (STAE) inhibited neuroinflammation and oxidative stress in A*β*_25‐35_‐induced Alzheimer's disease (AD) rats. (A) Cyclooxygenase‐2 (COX‐2) level (*n* = 6). (B) Tumor necrosis factor‐*⍺* (TNF‐*α*) level (*n* = 6). (C) NO synthesis (NOS) level (*n* = 6). (D) NO level (*n* = 6). (E) Interferon‐*γ* (IFN‐*γ*) level (*n* = 6). (F) Interleukin‐1*β* (IL‐1*β*) level (*n* = 6). (G) IL‐6 level (*n* = 6). (H) IL‐4 level (*n* = 6). (I) IL‐10 level (*n* = 6). (J) TGF‐*β* level (*n* = 6). (K) Reactive oxygen species (ROS) level (*n* = 6). (L) Malondialdehyde (MDA) level (*n* = 6). (M) Superoxide dismutase (SOD) activity (*n* = 6). (N) SOD1 activity (*n* = 6). (O) SOD2 activity (*n* = 6). (P) SOD3 activity (*n* = 6). (Q) Glutathione (GSH) activity (*n* = 6). (R) GSH‐Px activity (*n* = 6). STAE, *Lithocarpus polystachyus* Rehd. aqueous extract; sham, sham group; sham + STAE8, sham group administrated with 80 mg/mL STAE; A*β*_25‐35_, A*β*_25‐35_ group; A*β*_25‐35_ + STAE2, A*β*_25‐35_ group administrated with 20 mg/mL STAE; A*β*_25‐35_ + STAE4, A*β*_25‐35_ group administrated with 40 mg/mL STAE; A*β*_25‐35_ + STAE4, A*β*_25‐35_ group administrated with 80 mg/mL STAE; A*β*_25‐35_ + DON, A*β*_25‐35_ group administrated with 1 mg/kg donepezil. The data were expressed as mean ± SD. **p < 0.01 versus sham group; ^##^ *p* < 0.01 versus A*β*_25‐35_ group. [Color figure can be viewed at wileyonlinelibrary.com]

Additionally, to assess the impact of STAE on neuroinflammation and oxidative stress in rats induced by A*β*_25‐35_, the corresponding ELISA kits were utilized to measure the levels of inflammatory cytokines, oxidative indicators, and antioxidant enzymes. The findings indicated a significant increase in the levels of COX‐2, TNF‐*α*, NOS, NO, IFN‐*γ*, IL‐1*β*, and IL‐6. However, STAE clearly reduced these pro‐inflammatory cytokines (Figure 6A–G). Moreover, STAE also elevated the levels of the anti‐inflammatory cytokines, including IL‐4, IL‐10, and TGF‐*β* in AD rats following A*β*_25‐35_ (Figure 6H–J). In addition, there was a notable increase in oxidative stress markers, including ROS and MDA, in AD rats induced by A*β*_25‐35_ compared to the sham group, whereas STAE effectively counteracted these alterations (Figure 6K,L). Meanwhile, the activities of antioxidant enzymes including SOD and its subtypes SOD1‐3, GSH, and GSH‐Px were reduced in A*β*_25‐35_‐treated rats compared to the sham group, whereas STAE effectively reversed such trends (Figure 6M–R). The evidence implies that STAE can successfully prevent AD‐induced inflammation and oxidative stress.

## 4 DISCUSSION

The lack of significant progress in the treatment of AD can be attributed to its intricate pathogenesis. It is widely acknowledged that neuronal loss and cognitive impairments in AD are closely linked to oxidative stress and neuroinflammation. Consequently, targeting neuroinflammation and oxidative stress inhibition holds promise as a therapeutic approach for AD. Therefore, effective drugs for AD treatment should exhibit potent anti‐neuroinflammatory and antioxidant properties. Based on the findings of our prior investigation, it was determined that STAE possesses significant antioxidant and neuroprotective properties.^24^, ^26^ Given this background and the pharmacological attributes of STAE, we have undertaken a comprehensive examination of the therapeutic impacts of STAE on AD and its potential mechanism.

The current study provides encouraging evidence that STAE can effectively enhance learning and memory deficits caused by A*β*_25‐35_ in AD rats, as demonstrated by the MWM test, Y‐maze test, and NOR test. These findings suggest that STAE holds therapeutic potential for AD. Nevertheless, the precise and comprehensive mechanism underlying the therapeutic effects of STAE remains unclear. SIRT6 exerts a regulatory effect on NF‐κB expression through the deacetylation of H3K9Ac within the promoter region of NF‐κB target genes.^33^ This process results in the downregulation of pro‐inflammatory factors, ultimately inhibiting reducing the synthesis of NLRP3 inflammasome.^34^ Hence, it was hypothesized that the interaction between SIRT6/NLRP3 could potentially disrupt the pathological conditions associated with AD. Consistent with expectations, the administration of STAE resulted in an upregulation of SIRT6 protein expression via H3K9Ac deacetylation, as well as a downregulation of NLRP3 protein expression and its downstream pyroptosis‐related genes following AD insult. These findings indicate that STAE ameliorates learning and memory impairment in AD rats, at least partly, through mediating SIRT6/NLRP3 signaling pathway.

Emerging evidence demonstrates that the A*β* species can be identified by various pattern recognition receptors located on microglia, subsequently triggering inflammatory pathways that result in the activation of astrocytes.^19^, ^35^ Prolonged or excessive activation of microglial cells and astrocytes may generate cytokines, chemokines, and ROS, thereby maintaining an oxidative microenvironment.^36^ NO is produced owing to the pro‑inflammatory response, and NO is much more harmful under pathological conditions that involve the production of ROS. Furthermore, NO has been shown to activate both the constitutive and the inducible isoforms of COX, which is upregulated in brain cells under pro‑inflammatory conditions and is considered a marker of the progression of dementia in this disease.^37^ As expected, STAE significantly inhibited microglia and astrocyte activation. Subsequently, STAE reduced levels of COX‐2, TNF‐*α*, NOS, NO, IFN‐*γ*, IL‐1*β*, and IL‐6 and elevated the levels of IL‐4, IL‐10, and TGF‐*β*. The results revealed that STAE attenuated the overactivation of microglia and astrocytes by suppressing the expressions of inflammatory cytokines.

ROS is defined as a heterogeneous population of biologically active intermediation, which are byproducts of normal cellular metabolism and exhibit a twofold biological function.^7^ In essence, ROS plays a positive part under typical physiological circumstances, whereas an excess of ROS is associated with reduced cognitive abilities.^38^ MDA, a terminal product of lipid peroxides, may indirectly indicate the extent of cell damage and is crucial in the occurrence and progression of AD.^39^ There is a positive relationship between MDA levels and the extent of oxidative stress in the body, so it can be used as an indicator for detecting oxidative stress.^40^ Encouragingly, our findings showed that STAE decreased levels of ROS and MDA. Additionally, reports indicate that SOD can convert an intracellular superoxide anion into hydrogen peroxide (H_2_O_2_). The SOD family consists of three isotypes including SOD1, SOD2, and SOD3. Among them, SOD1 predominantly resides in the cytosol, while SOD2 and SOD3 are found in the mitochondrial matrix and the extracellular area, respectively. Our results indicated a notable increase in SOD1, SOD2, and SOD3 activities due to STAE, implying its primary role in removing ROS from the mitochondrial matrix, cell extracellular space, and cytosolic. Both GSH and GSH‐Px play crucial roles in the antioxidant mechanism. GSH, a tripeptide, exists in two forms including its reduced form (GSH) and oxidized form (GSSG).^41^ The GSH structure contains an active thiol group, which can eliminate free radicals through enzymatic or nonenzymatic catalysis. Additionally, it has the ability to create chelates with heavy metals and be expelled from the body.^42^ GSH‐Px is a prevalent enzyme in the body that breaks down peroxidase, primarily functions at selenocysteine.^43^ GSH‐Px is capable of facilitating the interaction between GSH and H_2_O_2_, breaking down lipid peroxides, converting harmful peroxides into harmless hydroxyls, aiding in H_2_O_2_ breakdown, and safeguarding cell membranes against harm.^44^ The results revealed that STAE apparently enhanced GSH and GSH‐Px activities, which suggests a parallel rise in substrate O_2_, H_2_O_2_, or peroxides, and a heightened MDA level further points to peroxidation in brain tissue.

The aforementioned findings imply that the protective effect of STAE is attributed to its anti‐neuroinflammatory and antioxidant properties. Of note, all redox elements were interconnected and regulated by various means, including antioxidant and vitagene network.^45^ Endogenous antioxidants such as NO and exogenous antioxidants such as naturally occurring polyphenols and flavonoids exhibit a hormetic dose responses to combat neurological disorders at low dose and lead to neurotoxicity at high dose.^46^, ^47^, ^48^ Hormetic dose responses is proposed that is according to the nuclear factor erythroid 2‐related factor 2 (Nrf2) and its upregulation of an endogenous antioxidant integrative system and anti‐inflammatory responses.^49^ Nrf2 is the main regulatory factor for antioxidant defenses, which maintains redox homeostasis through its downstream genes in cells/tissues by activating various vitagenes and other protective molecules.^50^ Hence, uncovering the interaction and coordination of redox interactions with endogenous and exogenous antioxidant defense systems is an emerging field of research interest in anti‐inflammatory anti‐degenerative therapeutics. According to results of our previous studies, the major active components of STAE display potent antioxidant activities with the activation of Nrf2 profile; we will explore whether STAE can also exert hormetic dose responses through the interaction between Nrf2 and vitagene network affect AD in our next story.

**Figure 7:**
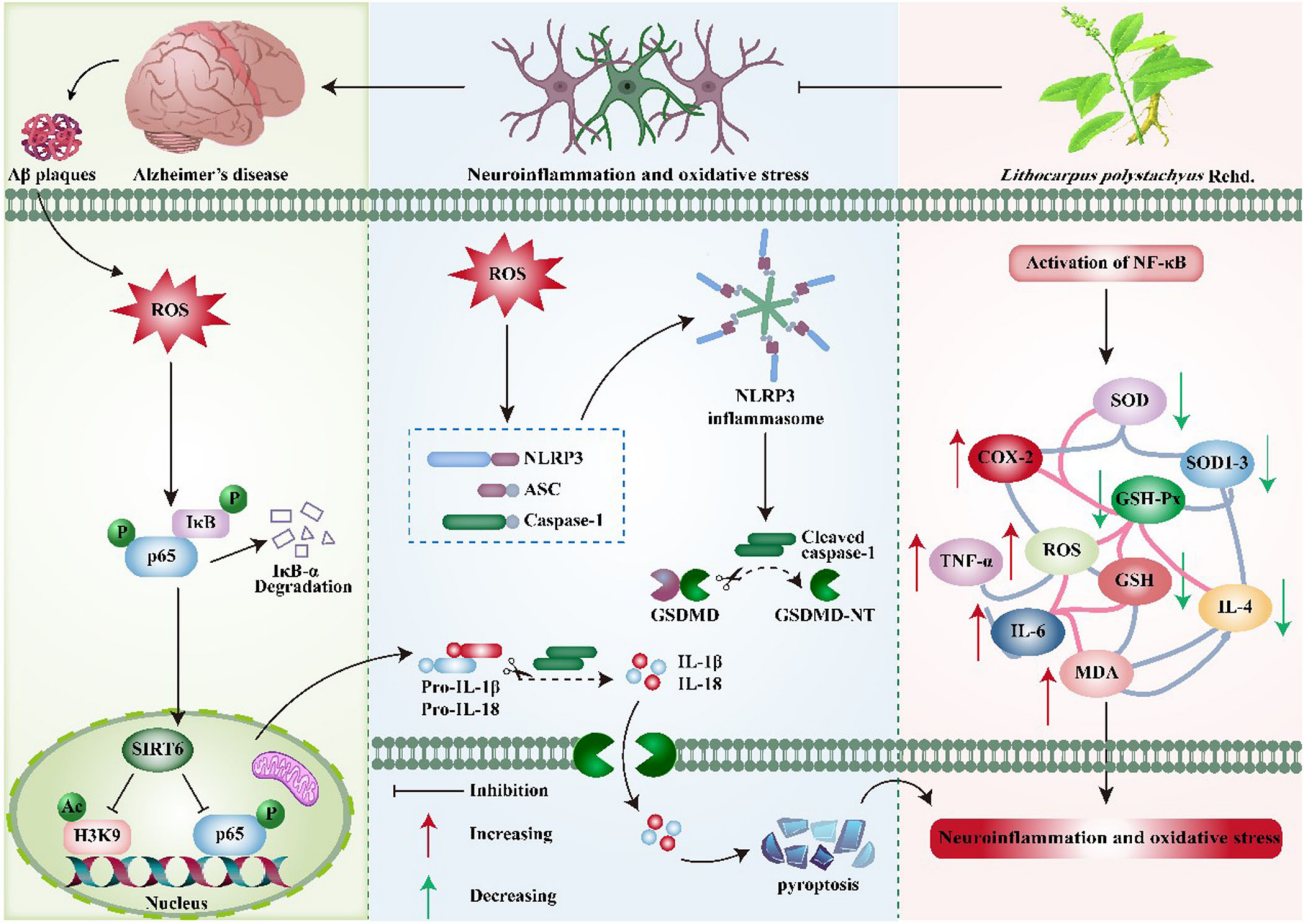
Schematic illustration of molecular mechanisms for the protective effect of Sweet tea aqueous extract (STAE) against Alzheimer's disease (AD). STAE may alleviate the neuroinflammation and oxidative stress induced by A*β*_25‐35_ via mediating the SIRT6/NLRP3 signaling pathway. [Color figure can be viewed at wileyonlinelibrary.com]

In conclusion, our findings indicate that STAE effectively mitigates A*β*_25‐35_‐induced cognitive decline in AD rats. This beneficial effect is attributed, at least in part, to the modulation of the SIRT6/NLRP3 signaling pathway. Consequently, it can be inferred that STAE holds potential as a valuable therapeutic intervention for the treatment of AD, and these results provide valuable insights into the potential therapeutic target of STAE for AD and offer a “proof‐of‐concept” for its efficacy in combating this disease (Figure 7).

## AUTHOR CONTRIBUTIONS

Wendan Wu, You Yan, and Tingting Yi performed the experiments. Yu Wei helped with IF analysis. Wendan Wu wrote the manuscript. Jianmei Gao and Qihai Gong designed the experiments and revised the manuscript. All authors were involved in the analysis of data and approved the final manuscript.

## CONFLICT OF INTEREST STATEMENT

The authors declare no conflict of interest.

## ETHICS STATEMENT

All experimental animal procedures in the present study were sanctioned by the Experimental Animal Ethics Committee of Zunyi Medical University (ZMU21‐2303‐333).

## Data Availability

The data sets generated and/or analyzed during the current study are available from the corresponding author upon reasonable request.
